# Supplementary material for: Complexome profiling of the Chlamydomonas psb28 mutant reveals TEF5 as an early PSII assembly factor
Source: Plant Cell. 2025 Mar 17;37(6):koaf055. doi: 10.1093/plcell/koaf055 (PMC12164590; doi:10.1093/plcell/koaf055)
Supplement: koaf055_Supplementary_Data [file koaf055_supplementary_data.zip › psb28_interactive.zip!/plots/intensity/qualityPlots/peptides/LHCA8_Cre06.g272650.t1.2.html]

### Cre06.g272650.t1.2

Trivial name: LHCA8  
SeqCoverage [median]: 71.60  
Mass: 25.92 kDa  
  
 

| Sequence | MissedCleavage | Mass [Da] | wtIntensity | muIntensity | Score |
| --- | --- | --- | --- | --- | --- |
| AGALNVPEWYDAGK | 0 | 1489.72 | 704504.57 | 800433.44 | 218.94 |
| DPWHVNYATNGVSLPFL | 0 | 1928.94 | 1746.83 | 0.00 | 100.93 |
| EADKWADWK | 1 | 1147.53 | 100394.86 | 61292.33 | 80.17 |
| GTSELGYPGGPFDPLGLSK | 0 | 1890.94 | 589521.39 | 579013.07 | 249.04 |
| KPGSQGEPGSFLGFEASLK | 1 | 1934.97 | 1044912.63 | 1963343.29 | 147.86 |
| KPGSQGEPGSFLGFEASLKGTSELGYPGGPFDPLGLSK | 2 | 3807.90 | 16483.85 | 0.00 | 78.19 |
| LAMLAFLGFVAQK | 0 | 1407.79 | 781062.55 | 534588.62 | 235.15 |
| NGRLAMLAFLGFVAQK | 1 | 1734.96 | 605.83 | 0.00 | 47.77 |
| QSWLPGSQIPAHLDTPAAQALAGNFGFDPLGLGKDPVALR | 1 | 4125.14 | 7949.75 | 0.00 | 65.38 |
| TAMAGVAGILIPGLLTK | 0 | 1624.96 | 447380.98 | 591588.18 | 297.05 |
| WQDIRKPGSQGEPGSFLGFEASLK | 2 | 2633.32 | 0.00 | 1311.09 | 43.89 |
| YATGAGPVDNLAAHLK | 0 | 1596.83 | 165317.04 | 326940.47 | 225.21 |
|  |  |  | 3859880.3 | 4858510.5 |  |
